# Comparing 3 Goal-Setting Techniques to Promote Adherence to National Physical Activity Guidelines in Midlife Adults: Feasibility Trial of a Mechanistic Study Design

**DOI:** 10.2196/82494

**Published:** 2026-03-16

**Authors:** Rodney P Joseph, Keenan A Pituch, Fang Yu, Dereck L Salisbury, Molly Maxfield

**Affiliations:** 1 Center for Health Promotion and Disease Prevention, Edson College of Nursing and Health Innovation, Arizona State University, 550 N 3rd Street, Phoenix, AZ, 85004, United States, 1 602-496-0772; 2 Edson College of Nursing and Health Innovation, Arizona State University, Phoenix, AZ, United States; 3 School of Nursing, University of Minnesota, Minneapolis, MN, United States

**Keywords:** physical activity, exercise, goal setting, midlife adults, Alzheimer disease, dementia, prevention

## Abstract

**Background:**

Engaging in regular aerobic physical activity (PA) during midlife is associated with reduced risk for Alzheimer disease and related dementias. Yet, most midlife adults fail to meet national PA guidelines. Goal setting is a commonly used behavior change technique to increase PA, but limited empirical evidence exists regarding whether certain types of goal setting are more effective than others. This study served as an initial step toward understanding how different goal-setting strategies may enhance PA and promote adherence to national PA guidelines among insufficiently active midlife adults with obesity.

**Objective:**

This study aimed to establish the feasibility and acceptability of a 4-arm mechanistic trial design comparing 3 different PA goal-setting techniques with a non–goal-setting comparison condition.

**Methods:**

This study was a 6-month stage IA mechanistic trial (based on National Institute on Aging’s Stage Model) that randomized insufficiently active midlife adults with obesity to 1 of 4 study groups: (1) Static Goal-Setting Group (weekly moderate to vigorous physical activity [MVPA] goal of 150 minutes), (2) Self-Selected Goal-Setting Group (participants self-selected a weekly MVPA goal), (3) Incremental Goal-Setting Group (weekly MVPA goal 20% greater than the previous week), and (4) Non–Goal-Setting Comparison Group (encouraged to increase MVPA without specific reference to a weekly goal). All participants (n=24) received a standardized, Social Cognitive Theory–based PA promotion intervention, which consisted of structured action planning sessions and a Fitbit activity monitor for self-monitoring of MVPA. The only difference across study groups was the goal-setting technique implemented. Primary outcomes included feasibility of study implementation, as assessed by recruitment, retention, and engagement rates; the ability to deliver the intervention as planned and collect outcomes necessary for evaluating the effects of different goal-setting techniques in a subsequent larger-scale trial; and participant acceptability of the intervention, as assessed by participant perceptions of and satisfaction with the intervention.

**Results:**

The sample (n=24; mean age 54.1, SD 5.8 years; mean BMI 36.3, SD 5.0 kg/m^2^) was recruited in approximately 4 months, equating to an enrollment rate of 6 participants per month. Retention at 6 months was 87.5% (21/24). Participants completing the intervention attended 85.9% of action planning sessions and wore the Fitbit for >10 hours per day on 87.1% of intervention days. Data collection rates for outcome measures ranged from 96% to 100%. Most participants expressed satisfaction with the intervention, with 86% (18/21) reporting gaining knowledge about PA and 90% (19/21) reporting that they would recommend the study to a friend.

**Conclusions:**

The next step in this research is to build on these findings by conducting a larger-scale phase 1B proof-of-concept trial to examine the preliminary effects of the goal-setting techniques for increasing MVPA and promoting adherence to national aerobic PA guidelines.

## Introduction

Alzheimer disease and related dementias (ADRD) are progressive neurodegenerative conditions that gradually erode memory and cognitive function. As ADRD progresses, individuals slowly lose the ability to independently perform routine activities of daily living (eg, bathing, dressing, preparing meals, and managing medications) and will require increased levels of care and support. Currently, more than 7 million Americans live with diagnoses of ADRD, an estimate that is projected to increase to approximately 14 million by 2060 [1]. Despite advancements in pharmacological treatments for ADRD in recent years, there are still no cures, underscoring the need for prioritizing risk reduction, particularly through targeting modifiable risk factors, which are estimated to contribute to approximately 45% of ADRD cases [2].

Regular aerobic physical activity (PA) is an established behavior associated with reduced risk for ADRD [2-5]. Aerobic PA is believed to lower risk for ADRD by modifying a number of ADRD pathogenic processes, including reducing AD β-amyloid load, tauopathy, neurodegeneration, and oxidative stress, and increasing neurogenesis and synaptogenesis, brain-derived neurotrophic factors, and antineuroinflammation [6,7]. In addition to these neuroprotective effects, PA is a behavioral cornerstone for the prevention and management of cardiometabolic diseases (obesity, hypertension, and diabetes) [8], which are also independently associated with increased risk for ADRD [2,6,9].

Although the cognitive health benefits of regular PA are evident across the lifespan, it is especially important during midlife [2,4,10]. Maintaining high PA levels during this stage of life has consistently shown to lower ADRD risk, even among individuals who were previously sedentary [3,10]. Despite this, only half of US adults aged 45‐65 years meet national PA guidelines for aerobic activity (ie, 150 minutes per week of moderate-intensity PA, 75 minutes per week of vigorous PA, or an equivalent combination of both moderate- and vigorous-intensity PA) [11]. Given the strong link between midlife PA and the risk of ADRD later in life, intervening to increase PA among insufficiently active individuals during midlife has potential to attenuate and reduce ADRD risk.

Previous interventions promoting PA during midlife have generally yielded modest and short-lived increases in PA [12,13]. Even among interventions that have successfully increased PA, most participants do not achieve or maintain PA levels consistent with national aerobic PA guidelines [12]. The continued inability of PA interventions to promote sustained increases in PA and adherence to national PA guidelines underscores the need for researchers to critically examine the behavior change techniques used in their intervention efforts, including those that appear to be effective, at least in the short term. Such analyses should pay particular attention to examining the psychosocial processes targeted by the behavior change techniques used, as failure to effectively engage or modify these underlying processes likely contributes to the modest impact and poor long-term adherence observed in many PA interventions. Understanding and addressing these mechanisms are essential to developing more effective interventions for promoting PA during midlife.

Goal setting is one of the most widely used behavior change techniques to increase PA. It is endorsed by leading authorities, including the National Physical Activity Guidelines for Americans [14] and the American College of Sports Medicine [15], and is a common feature incorporated into commercial PA trackers (ie, Fitbit, Garmin, and Apple Watch devices). Meta-analyses support the effectiveness of goal setting for increasing PA among participants of all ages [16,17], although long-term adherence to change is not well documented. Although goal setting appears to be an effective short-term strategy to increase PA, there is no clear consensus about how goal setting should be implemented to increase PA to support long-term engagement. This is evidenced by substantial heterogeneity in goal-setting techniques used by researchers [16] and ongoing debate in the peer-reviewed literature [18,19]. The variability and inconsistency in goal-setting techniques implemented by researchers may explain, in part, why most PA interventions fail to consistently result in participants achieving and maintaining high PA levels. Moreover, few studies have examined why different types of goal-setting techniques may be more effective for increasing PA and maintaining high PA levels than others [17].

The National Institutes of Health (NIH) Science of Behavior Change Program [20] has recommended the use of an experimental medicine approach to gain a better understanding of how and why behavior change occurs. This approach focuses on the identification and explicit testing of the underlying psychosocial processes (referred to as mechanisms) believed to underpin the use of specific behavior change techniques. In the context of PA, goal setting is thought to influence key psychosocial mechanisms of self-regulation (ie, ability to manage social, cognitive, and motivational processes to increase PA) and self-efficacy (ie, confidence or perceived ability to overcome barriers and increase PA) [15,21-24]. However, the literature lacks robust evidence on how specific goal-setting techniques influence these mechanisms, underscoring the need for mechanistic trials to empirically examine how PA goal setting can be best implemented to enhance these psychosocial processes for successful promotion of PA.

This study represented an initial step toward better understanding how different types of goal-setting techniques influence psychosocial mediators of self-regulation and self-efficacy, as well as PA and adherence to national PA guidelines. Specifically, we examined the feasibility and acceptability of a mechanistic study designed to compare 3 distinct goal-setting techniques and a non–goal setting comparison group for enhancing psychosocial constructs of self-regulation and self-efficacy and for promoting adherence to national aerobic PA guidelines. Focal areas of evaluation included the following: (1) Feasibility of study implementation, as assessed by the research team’s ability to recruit, retain, and engage participants in the intervention; deliver the intervention as planned; and collect outcome data necessary for evaluating the effect of different goal-setting techniques on self-regulation, self-efficacy, and PA in a subsequent larger-scale trial. (2) Participant acceptability of the intervention, as assessed by participants’ perceptions of and satisfaction with the intervention.

## Methods

### Overview

This study was conducted as part of an NIH/National Institute on Aging–funded R61/R33 phased award. The R61 phase of the award focused on intervention development and feasibility testing through conducting a stage IA feasibility trial to inform intervention refinement in preparation for larger-scale testing in a stage IB proof-of-concept trial in the R33 phase, which will evaluate the effects of goal-setting techniques to increase PA, promote adherence to national PA guidelines, and enhance psychosocial constructs of self-regulation and self-efficacy. This paper reports the design and outcomes of a stage IA trial conducted as part of the R61 phase of the award. Milestones for progression from the R61 to R33 phase of the award included (1) development of all intervention materials, (2) conducting the stage IA trial (24 participants), and (3) obtaining participant feedback for refinement of the experimental approach for subsequent testing in a stage IB trial. Accordingly, the study design and feasibility outcomes of the current trial were aligned with these progression milestones, rather than selection and achievement of predefined quantitative metrics of feasibility and acceptability to justify advancement to the next stage of research.

### Study Design

**Figure 1.**
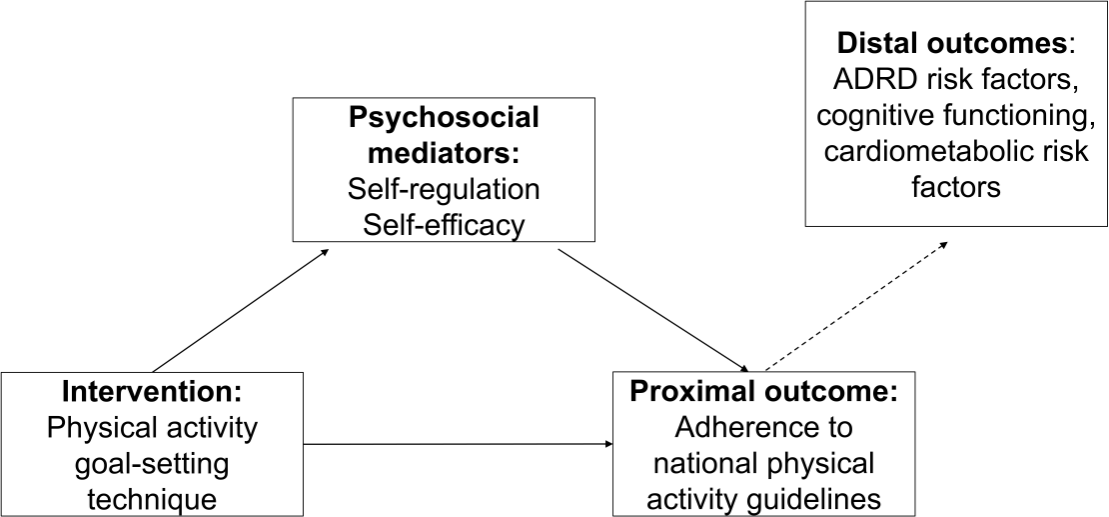
Illustration of the conceptual mediation model underpinning the design of the study. ADRD: Alzheimer disease and related dementias.

Guided by the NIH Stage Model [25] and the feasibility framework of Bowen et al [26], a 6-month stage IA feasibility trial was conducted using a randomized, 4-arm mechanistic study design. All participants (n=24) received a standardized, Social Cognitive Theory (SCT)–based PA promotion intervention and were randomly assigned to 1 of the 3 goal-setting conditions or a non–goal setting comparison group (Table 1). This 4-arm study design was selected because when implemented in a fully powered trial, it will enable attribution of any observed difference in self-regulation, self-efficacy, and PA across study arms to the specific goal-setting technique tested in each group. Figure 1 illustrates the conceptual model underpinning the design of the study.

**Table 1.** Description of the 3 goal-setting experimental conditions and the non–goal-setting comparison condition tested in the 4-arm feasibility trial.

Goal-setting technique	Description	Rationale of inclusion in the proposed trial
Static weekly MVPA^a^ goal of 150 minutes	Participants were assigned a fixed weekly MVPA goal of 150 minutes per week throughout the intervention. Each action planning session focused on the participant achieving this weekly goal.	This approach represents the straightforward recommendation of public health campaigns and health care providers and is a common PA^b^ goal recommended in many PA promotion interventions.
Self-selected weekly MVPA goal	Participants self-selected their own minute-per-week MVPA goal each week. The goal could be any number of minutes, regardless of whether it was above, equal to, or below the national PA guidelines. Action planning sessions focused on helping participants achieve their self-selected goal.	This approach relies on the level of self-motivation to achieve the goal and incorporates participant autonomy. Self-selected goals have proven effective in both PA [16] and non–PA-related goal achievement [27]. This approach has also been suggested as ideal, particularly among individuals with low PA levels, because goals will be personally relevant, allow for flexibility, and account for priorities and time factors [16,18].
Incremental weekly MVPA goal increase	Participants were assigned a unique minute per week MVPA goal each week that was 20% greater than the minutes of MVPA performed the previous week, regardless of whether they achieved the incremental weekly goal assigned the prior week. Action planning sessions focused on the participant achieving this personalized goal.	This approach aligns with Locke and Latham’s [28] goal-setting theory and tenets of SCT^c^, using a gradual increase in steps to achieve national standards and allowing for steady adjustment to change in activity. Designed to make goals more achievable, this approach is predicted to lead to greater levels of self-efficacy, self-regulation, and adherence over time.
No stated MVPA goals (comparison condition)	Action planning sessions did not include explicitly stated MVPA goals. Sessions focused on increasing PA, in general, without mention of achieving a weekly PA goal.	This condition served as the comparison (or control) condition. Participants may have generated their own goals, but they were not addressed by the interventionist during PA action planning sessions. Inclusion of this study group allows for differences in PA outcomes across study groups to be attributed to the goal-setting techniques tested. This comparison group was selected instead of a wellness control or delayed intervention group because it allows for experimental testing of the effects of not using goal setting within the context of the broader SCT-based intervention provided to all participants.

a MVPA: moderate to vigorous physical activity.

b PA: physical activity.

c SCT: Social Cognitive Theory.

### Ethical Considerations

This study was reviewed and approved by the institutional review board of Arizona State University (STUDY00016394) and was registered with ClinicalTrials.gov (NCT05980052) within 30 days of enrolling the first participant. All participants provided written informed consent and were informed of their right to withdraw from the study at any time. Study data were deidentified prior to data analysis. Participants received up to US $120 in compensation (US $20 for completing the study orientation, US $50 for completing the baseline assessment, and US $50 for completing the 6-month follow-up assessment) and were allowed to keep their Fitbit Versa 2. Study methods and outcomes are reported in accordance with the CONSORT (Consolidated Standards of Reporting Trials) guidelines for randomized pilot and feasibility trials [29].

### Sample Size Considerations

An a priori sample size of 24 participants was informed by recommended best practices for the design of pilot studies [30,31] and based on the research team’s determination that it would provide sufficient data for evaluation of the primary aims for this phase of the research study, which were to establish the feasibility and acceptability of the scientific approach. Given that the study was not designed to assess within or between-group intervention effects, no formal power calculations were conducted.

### Study Setting and Timeline

All study activities were conducted on the Downtown Campus of Arizona State University, located in Phoenix, Arizona. Recruitment occurred between June 2023 and October 2023. The first participant enrolled in the study on July 17, 2023, and the last participant completed all study-related activities on May 13, 2024, which concluded data collection for the pilot trial.

### Recruitment

Community-based strategies were used to recruit participants. These strategies included email distribution lists, paid social media advertisements (ie, Facebook and Twitter), flyers posted at community locations, and word-of-mouth referrals.

### Participants

Study participants (n=24) were insufficiently active midlife adults with obesity (Textbox 1).

Textbox 1. **Inclusion and exclusion criteria.**

**Inclusion criteria**

- Age 45-65 years.
- BMI between 30 kg/m^2^ and 50 kg/m^2^.
- Engaging in 60 minutes per week or less of self-reported moderate to vigorous physical activity at screening (based on Exercise Vital Sign Questionnaire [32]).
- Self-reported ownership of a smartphone with an iOS or Android operating system.

**Exclusion criteria**

- Endorsing an item on the Physical Activity Readiness Questionnaire [33] indicating contraindication to moderate to vigorous physical activities, unless written clearance from their health care provider for participation in the study was provided.
- Resting blood pressure greater than 200/110 mm Hg, as assessed at the baseline study assessment.
- Pregnant or plans to become pregnant in the next 8 months.
- Plans to relocate out of the metropolitan Phoenix, Arizona, area in the next 8 months.
- Participation in another physical activity, nutrition, or weight loss program at the time of screening or at any time during the intervention.
- Previous diagnosis of mild cognitive impairment (MCI) or a score indicative of MCI on the Montreal Cognitive Assessment [34], defined as a score of <26 for US-born participants or <23 for those who completed school in a country where English is not the primary language, as assessed during the study orientation.
- Being previously prescribed 1 of the 5 approved Alzheimer disease medications, including donepezil (Aricept), rivastigmine (Exelon), galantamine (Razadyne), memantine (Namenda), and memantine + donepezil (Namzaric).
- Scoring ≥16 on the Center for Epidemiological Studies Depression Scale.
- Current diagnosis of major depression.
- Currently taking 2 or more antidepressant drugs.
- Self-reported history of stroke.

### Intervention Description

#### Overview

All participants received a standardized, SCT-based [22] PA promotion intervention that consisted of regular action planning sessions with a trained interventionist and a Fitbit Versa 2 activity monitor to wear daily to self-monitor their moderate to vigorous PA (MVPA). The only difference among the study groups was the specific type of PA goal participants were encouraged to achieve throughout the intervention, or the absence of goal setting in the comparison group. Additionally, all participants received a study handbook outlining the importance of PA for physical, mental, and cognitive health, along with tips for safely increasing activity and overcoming common barriers.

#### Theoretical Framework

SCT was selected as the theoretical basis for the standardized intervention delivered to study participants because it explains behavior using a dynamic and reciprocal model in which individual factors (beliefs and attitudes), the social and physical environments, and behavioral factors interact [22]. Given PA is largely influenced by an individual’s thoughts, beliefs, attitudes, perceived control over their behaviors, and their social environment, SCT provided a meaningful framework to guide the PA action planning component of the intervention. Table 2 provides an overview of the SCT constructs leveraged by the intervention, as well as the specific behavior change techniques implemented in the action planning intervention based on each study group.

**Table 2.** Overview of how the standardized intervention delivered to all study participants leveraged constructs of Social Cognitive Theory and established behavior change techniques to promote physical activity.

SCT^a^ construct	Description of how the intervention targets each theoretical construct	Behavior change techniques employed based on Michie’s Taxonomy (v1)^b^		Study group in which the behavior change technique was employed^c^			
		No.	Label	Static	Self-Selected	Incremental	Non-Goal
Behavioral Capability - knowledge and skill to perform a PA^d^	PA action planning materials and sessions provided information on the definitions of PA and exercise, different types of PA (aerobic vs. muscle strengthening), national PA guidelines, modes/types of PA that can be performed to achieve PA guidelines, how to determine PA intensity.	4.1	Instruction on how to perform a behavior	✓	✓	✓	✓
Outcome Expectations - Anticipated outcomes of PA	Action planning sessions provided participants with health and social outcomes associated with being physically activity, including reduced risk for ADRD^e^, cognitive decline, heart disease and type 2 diabetes, weight maintenance, more energy to perform daily activities, improved quality and quantity (i.e., years) of life, being a good role model to family/friends.	5.1	Information about health consequences	✓	✓	✓	✓
		5.3	Information about social and environmental consequences				
		5.6	Information about emotional consequences				
	Action planning sessions had participants identify the personally relevant anticipated outcomes for increasing MVPA^f^.	9.2	Pros and cons	✓	✓	✓	✓
		13.4	Valued self-identity				
		13.5	Identity associated with behavior change				
Self-regulation- Ability to manage social, cognitive, and motivational processes to achieve a desired PA goal	Goal setting: Participants were encouraged to achieve their study-group specific weekly MVPA goal to ultimately achieve 150 min/week of moderate-intensity PA by end of the active 6-month intervention. During action planning sessions, the PA coach and participant jointly reviewed progress toward goal achievement and strategies to achieve the study-specific goal for the next week.	1.1	Goal setting (behavior)	✓	✓	✓	×
		1.5	Review of behavioral goal(s)				
		1.6	Discrepancy between current behavior and goal				
		2.2	Feedback on behavior				
	Self-monitoring: Participants used Fitbit to self-monitor MVPA.	2.1	Self-monitoring of behavior	✓	✓	✓	✓
	Self-reward: Interventionists encouraged participants in the static, self-selected, and incremental goal setting groups to create self-rewards for achieving MVPA goals. Participants in the non-goal setting group were encouraged to select self-rewards for PA behaviors in general, without reference to minutes/week of MVPA.	10.7	Self-incentive	✓	✓	✓	✓
		10.9	Self-reward				
	Barrier problem solving: Participants were encouraged to identify personal barriers to MVPA and engage in problem solving discussions with action planning interventionist to overcome barriers.	1.2	Problem solving	✓	✓	✓	✓
		1.4	Action planning				
		15.3	Focus on past successes				
	Lapse/Relapse Prevention: Interventionists monitored Fitbit data to identify MVPA lapses and work with participants to identify reasons for lapses and to help overcome them in the future.	1.2	Problem solving	✓	✓	✓	✓
Self-efficacy - Confidence in oneself to take action and overcome barriers	Mastery Experiences: Participants enact strategies identified during action planning sessions to increase MVPA and track their increases/decreases using the Fitbit and app activity tracking feature.	8.1	Behavioral practice/ rehearsal	✓	✓	✓	✓
		8.6	Generalization of a target behavior				
	Social Modeling: Action planning interventionists provided participant with strategies they utilize to be physically active and encourage participants to identify individuals in their own life they can look-up to as PA role models.	6.1	Demonstration of the behavior	✓	✓	✓	✓
		6.2	Social comparison				
	Verbal Persuasion: Action planning interventionists provided words of encouragement and empowerment for PA and provided individualized feedback/encouragement during action planning sessions.	15.1	Verbal persuasion about capability	✓	✓	✓	✓
		3.2	Social support (practical)				
		3.2	Social support (emotional)				
	Emotional arousal: Action planning sessions reinforced the notion that PA doesn’t have to be structured or difficult by encouraging walking and by providing tips on how participants can incorporate more PA into the day.	15.1	Verbal persuasion about capability	✓	✓	✓	✓

a SCT: Social Cognitive Theory.

b Behavior change techniques are coded based on Taxonomy of Behavior Change (version 1) by Michie et al [35].

c Check marks (✓) indicate that the behavior change technique(s) used to target the SCT construct were included in the study group’s action planning sessions. Cross mark “X” mark indicates that the behavior change technique(s) were not included in the study group’s action planning sessions.

d PA: physical activity.

e ADRD: Alzheimer disease and related dementias

f MVPA: moderate to vigorous physical activities.

In addition to SCT, Locke and Latham’s Goal Setting Theory [28] guided the selection of the incremental goal-setting study group (Table 1). This theory posits that setting specific, challenging, yet achievable goals, combined with constructive feedback, enhances motivation and self-efficacy for achieving a goal, ultimately leading to behavior change to support goal attainment.

#### Goal Setting Experimental Conditions

Table 1 provides a detailed description and the underlying rationale for inclusion of each experimental condition. The three goal-setting experimental conditions included (1) static weekly goal, where participants were encouraged to meet a fixed weekly MVPA goal of 150 minutes; (2) self-selected weekly goal, where participants were encouraged to create their own weekly MVPA goals; and (3) incremental weekly goal, where participants were assigned a weekly MVPA goal 20% greater than the previous week’s goal. In addition to these 3 goal-setting conditions, a non–goal-setting comparison group was included, in which participants were encouraged to increase MVPA without setting specific weekly goals.

Selection of the goal-setting techniques was informed by a comprehensive review of the literature on the types of PA goals commonly incorporated into PA promotion interventions [16], as well as a review of the theoretical assumptions underpinning the use of goal setting [18,19]. Minutes per week of MVPA was chosen as the behavioral target for all goal-setting study groups to align with the metric used in the national PA guidelines [14]. By directly comparing multiple commonly used goal-setting techniques and a non–goal-setting comparison condition within a single experimental framework, this study addresses an important gap in the literature, as few studies have systematically evaluated multiple goal-setting approaches to support midlife adults in meeting national PA guidelines.

#### Standardized SCT-Based Intervention

Over the duration of the 6-month intervention, participants engaged in 17 structured action planning sessions, which were referred to as “coaching sessions” by the research team for all participant-facing communications. These sessions were conducted via telephone or Zoom (Zoom Communications, Inc) videoconferencing platform, based on participant preference. Session frequency occurred weekly during months 1‐3, every other week during months 4 and 5, and once during month 6. The gradual withdrawal of participant contact with study interventionists was designed to promote autonomy and encourage participants to rely on their own self-regulatory and self-efficacy processes promoted through their study arm–specific goal-setting technique to achieve weekly PA goals and ultimately national PA guidelines.

Table 3 illustrates the structured content of the action planning sessions. The first 2 sessions were designed to be approximately 1 hour in duration and focused on the interventionist working with the participant to develop an individualized action plan for increasing their PA. Sessions 3‐17 were designed to be approximately 15 minutes in duration and consisted of reviewing the participant’s PA progress since the last session, discussing PA successes and areas for improvement, engaging in PA barrier problem solving, discussing their next weekly PA goal (based on study arm allocation; participants in the non–goal-setting comparison group did not discuss PA goals), and identifying psychosocial and behavioral strategies for the participant to achieve their next PA goal. The action planning sessions were conducted by trained research staff, all of whom completed study-specific training led by the study investigators (RPJ and MM) before engaging with participants. Interventionists followed a manualized protocol when delivering coaching sessions and documented session activities and other relevant details about the session (eg, participant responses and reactions to prompts) in real time via the study’s REDCap (Research Electronic Data Capture; Vanderbilt University) database.

**Table 3.** Overview of the action planning session activities completed by participants across the 4 study groups as part of the standardized Social Cognitive Theory–based intervention.

Activity			Social Cognitive Theory and goal-setting^a^ constructs targeted	Approximate duration
Session 1: Action planning activities				
I. Greetings and initial introductions.			N/A^b^	5 minutes
II. Review the purpose of the study.			N/A	5 minutes
III. Presentation of the national aerobic PA^c^ guidelines and the different types and modes of PA that can be performed to achieve national aerobic PA guidelines.			Behavioral capability	10 minutes
IV. Overview of the health and social benefits of being physically active and achieving the national PA guidelines.			Outcome expectations and goal setting	5 minutes
V. Participant identifies reasons they want to be physically active and the types of aerobic PA they are interested in performing (ie, walking, group exercise classes, and cycling).			Outcome expectations	5 minutes
VI. Assignment of first weekly moderate-intensity PA goal (based on study arm); non–goal setting study arm did not engage in this activity.			Self-regulation and goal setting	1‐2 minutes
VII. Participant identifies non–PA-related previous life experiences where they worked hard to achieve a goal they set out for themselves; the interventionist helps the participant identify effective strategies used to achieve the goal.			Self-regulation, self-efficacy, and goal setting	5‐10 minutes
VIII. Review of common PA barriers; participant identifies PA barriers they anticipate facing and engages in problem-solving strategies with the interventionist to overcome anticipated barriers.			Self-regulation and self-efficacy	10 minutes
IX. Participant and interventionist brainstorm and identify behavioral strategies to incorporate PA into their day to achieve their study group–specific weekly PA goal, including specifying the types of PA participants would engage in to achieve their PA goal; non–goal-setting group focused on general strategies to increase their PA without reference to achieving a specific goal.			Self-regulation	5‐10 minutes
X. Interventionist and participant schedule a time for next action planning meeting.			N/A	1 minute
Approximate duration of initial action planning session				1 hour
Session 2: Action planning activities				
I. Greetings and pleasantries.			N/A	1‐2 minutes
II. Review of the purpose of the study, minutes per week of MVPA^d^ performed since the previous action planning session, and progress toward achievement of previous week’s PA goal; non–goal-setting group reviews minutes per week of MVPA without reference to any goal.			N/A	5 minutes
III. Participant and interventionist discuss the psychosocial and behavioral strategies the participant performed since the last action planning sessions to achieve their study group–specific PA goal; non–goal-setting group focuses on strategies used to increase their activity, without mention of a specific goal. Interventionists provide positive reinforcement for successfully implemented strategies.			Self-efficacy	5 minutes
IV. Interventionist provides an overview of self-rewards for achieving PA goals and the importance of social support when trying to achieve physical activity goals; non–goal-setting group focuses on self-rewards for being physically active, in general, and the importance of social support for increasing PA.			Self-regulation	10 minutes
V. Participant and interventionist collaboratively develop self-rewards for the participants achieving their short- and long-term PA goals; non–goal-setting group focuses on self-rewards for being physical activity in general, without mention of any specific goals. Discussion of the importance of social support.			Goal setting and self-regulation	10 minutes
VI. Participant and interventionist collaboratively identify people in the participant’s life who can serve as sources of social support for PA.			Social support	10 minutes
VII. Participant identifies 3 PA barriers faced since last session, and strategies to overcome them during the next week, including specification of the types of PA they intend to engage in during the upcoming weeks.			Self-regulation and Self-efficacy	10 minutes
VIII. Assignment of PA goal for the next week.			Self-regulation and goal setting	5 minutes
IX. Interventionist and participant schedule a time for next action planning meeting.			N/A	1 minute
Aproximate duration of action planning session 2				1 hour
Sessions 3‐17: Action planning activities				
I. Greetings and pleasantries.			N/A	1 minute
II. Review of PA goal(s) since last action planning session and progress toward goal achievement.			Goal setting	1 minute
III. Participant and interventionist discuss the psychosocial and behavioral strategies the participant performed since the last action planning sessions to achieve their study arm specific PA goal; interventionist provides positive reinforcement for successfully implemented strategies.			Self-efficacy	2‐3 minutes
IV. Discussion of PA obstacles or barriers the participant faced since last action planning session.			Self-regulation and self-efficacy	2‐3 minutes
V. Assignment or discussion of new weekly or biweekly moderate-intensity PA goal (based on study arm).			Goal setting	1‐2 minutes
VI. Participant and interventionist discuss and identify strategies to overcome previously identified or anticipated PA barriers for the upcoming week(s), including encouraging the participant to specify the types of PA they intend to engage in to achieve their PA goal during the upcoming week; non–goal-setting group includes this activity but focuses on increasing overall PA, rather than achieving a specific goal.			Self-regulation and self-efficacy	2‐3 minutes
VII. Reinforcement of the participant’s value-based reasons they want to increase their PA (assessed during the initial action planning session).			Self-efficacy	2‐3 minutes
VIII. Interventionist and participant schedule a time for next action planning meeting.			N/A	1 minute
Estimated duration of action planning sessions				12‐17 minutes

a Participants in the non–goal-setting comparison condition did not engage in any goal-setting activities in sessions**; **coaching sessions focused on increasing PA in a general sense without reference to any specific goal.

b N/A: not applicable.

c PA: physical activity.

d MVPA: moderate to vigorous physical activities.

To facilitate self-monitoring of MVPA and to track progress for weekly MVPA goal achievement, participants were given a Fitbit Versa 2 activity monitor and asked to wear it 24 hours per day, except when charging. Fitbit’s metric of “Active Minutes” was originally intended to serve as the self-monitoring measure of MVPA. However, during the early stage of study implementation, Fitbit modified the terminology and propriety algorithms used to assess MVPA and transitioned to use of “Active Zone Minutes,” which subsequently served as the self-monitoring measure of MVPA following this change. All Fitbit accounts were preregistered to the research team to allow the team to troubleshoot participant-related Fitbit issues during the intervention and were connected to the Fitabase data collection management platform (Small Steps Lab LLC). This allowed the research team to remotely monitor and track participants’ PA levels throughout the intervention.

Several steps were taken to ensure participant comprehension of what constitutes MVPA and how it feels to perform MVPA. First, during the baseline study assessment procedures, participants walked at a moderate intensity on a treadmill at 0% grade during the warm-up phase (based on age-estimated heart rate) of the cardiopulmonary exercise test (Table 4). Study staff explicitly referenced this walking pace and the associated physiological sensations (eg, heart rate and breathing) as the reference point for participants to gauge moderate-intensity PA throughout the study. In addition, coaching session 1 provided an overview of the types of activities that constitute MVPA and reviewed various methods for gauging exercise intensity, including heart rate–based monitoring using their Fitbit device, the “talk test” (eg, during moderate-intensity exercise, individuals should be able to talk but not sing; during vigorous-intensity activity, they should not be able to say more than a few words without taking a breath), and using a 1‐10 perceived exertion scale, where moderate intensity corresponds to a rating of 5‐6 and vigorous intensity corresponds to a rating of 7 or higher.

**Table 4.** Key measures identified for inclusion in a subsequent larger-scale trial designed to evaluate the effects of different goal-setting techniques to enhance self-regulation and self-efficacy and adherence to national aerobic physical activity guidelines.

Measures	Timepoint collected	
	Baseline	6 months
Psychosocial mediators: self-efficacy and self-regulation		
Physical Activity Self-Regulation Scale (PASR-12) [21]: This 12-item scale assesses 6 PA^a^-related self-regulatory domains: self-monitoring, goal setting, eliciting social support, reinforcements, time management, and relapse prevention. The PASR-12 has established structural and construct validity, as well as interrater reliability [21].	Monthly	
Self-Regulation Scale from the Health Belief Survey [36]: This 10-item questionnaire assesses behavior strategies to incorporate PA into daily activities (ie, take stairs instead of elevator and walking instead of driving when running errands). This questionnaire has been validated and has acceptable reliability estimates in previous research (Cronbach α range: 0.64-0.74) [36,37].	Monthly	
Short Self-Regulation Questionnaire [38,39]: The 33-item Short Self-Regulation Questionnaire measures ability to regulate behaviors for goal pursuit. Publicly available in the NIH Science of Behavior Change repository, the measure has established validity and reliability (Cronbach α=0.92) [38,39].	Monthly	
Exercise Self-Efficacy Scale [40]: This 12-item questionnaire assesses confidence in one’s ability to perform PA despite barriers or obstacles. This measure has established validity and adequate reliability (Cronbach α=0.78) [40].	Monthly	
Self-Efficacy Scale [41]: The 10-item questionnaire assesses beliefs in one’s capacity to manage daily stressors and exert control in daily life. Publicly available in the NIH Science of Behavior Change repository, the NIH Toolbox [42] has established validity and reliability (Cronbach α=0.94) [41].	Monthly	
Proximal study outcomes: physical activity		
Objective Assessment of Physical Activity: Assessed using ActiGraph GT9X Link activity monitors worn on the nondominant wrist during waking hours for 7 consecutive days. Valid wear is defined as wearing the ActiGraph for >10 waking hours per day during the 7-day wear period based on the Choi algorithm [43] to determine nonwear time.	✓	✓
Subjective Assessment of Physical Activity: Assessed by the 7-Day Physical Activity Recall (7-Day PAR) [44]. This interviewer-administered instrument assesses self-reported minutes per week of MVPA^b^. The 7-Day PAR has established reliability among older adults [45] and has been validated against accelerometers for accurate assessment of PA [46].	✓	✓
Distal study outcomes: measures of cognitive functioning, ADRD^c^, and cardiometabolic disease risk		
Episodic Memory: Picture Memory Test [47] from the Cognitive Assessment section of the NIH Toolbox for the Assessment of Neurological and Behavioral Function.	✓	✓
Executive Functioning: Dimensional Change Card Sort Test [48] from the Cognitive Assessment section of the NIH Toolbox.	✓	✓
Processing Speed: Pattern Comparison Processing Speed Test [49] from the Cognitive Assessment section of the NIH Toolbox for the Assessment of Neurological and Behavioral Function.	✓	✓
Serum and plasma measures of ADRD and Cardiometabolic Disease Risk: Assessed by standardized phlebotomy and blood-processing procedures collected after 8 hours of fasting. Measures of ADRD risk included plasma amyloid 42/40 ratio and plasma phosphorylated tau 231. Measures of cardiometabolic disease risk included fasting plasma glucose, serum insulin, and serum lipids (TG^d^, total cholesterol, LDL^e^—cholesterol, and HDL^f^—cholesterol).	✓	✓
Cardiorespiratory Fitness: Assessed using a modified Blake treadmill protocol. Testing and termination criteria followed ACSM^g^ guidelines.	✓	✓
BMI: Measured using an electric scale and calculated using the formula: weight (in kilograms) divided by height (in meters) squared.	✓	✓
Waist circumference: Measured at midpoint between rib and top of iliac crest.	✓	✓
Blood pressure: Assessed in triplicate following a 5-minute rest using an electronic sphygmomanometer.	✓	✓

a PA: physical activity.

b MVPA: moderate to vigorous physical activities.

c ADRD: Alzheimer disease and related dementias.

d TG: triglycerides.

e LDL: low-density lipoprotein.

f HDL: high-density lipoprotein.

g ACSM: American College of Sports Medicine.

### Protocol

Individuals interested in participating in the study completed an online eligibility screening survey administered via REDcap. Those meeting initial eligibility criteria were contacted by the research team and completed a telephone screening interview to verify initial eligibility. Those who met the eligibility criteria and remained interested were invited to attend an in-person orientation session. During this session, a research team member reviewed the informed consent form, addressed any questions, and obtained written informed consent from participants who chose to proceed. Following consent, the research team measured participants’ height and weight to confirm eligibility based on BMI and administered the Montreal Cognitive Assessment (MoCA) [34] and the Center for Epidemiologic Studies Depression Scale [50] to screen for cognitive impairment and depression, respectively. Participants who remained eligible after these assessments were scheduled for their baseline study visit, provided an ActiGraph GT9X Link accelerometer to wear for 7 days for objective assessment of their PA levels, and emailed the baseline survey measures (Table 4).

Following the 7-day accelerometer wear period, participants attended their baseline assessment visit. During this visit, preintervention data were collected, including anthropometric measures, phlebotomy, cognitive testing, self-reported PA levels via the 7-Day Physical Activity Recall [44], and a cardiopulmonary exercise test (Table 4). Once all baseline data were collected, the participant was randomized by a member of the research team using REDCap’s randomization module. Stratified block randomization, using blocks of size 4, with strata biological sex (male and female) and obesity disease status (BMI 30 to <40 kg/m^2^ [ie, class 1 and 2 obesity]; BMI >40 kg/m^2^ [ie, class 3 obesity]), was performed. This approach ensured that treatment groups were balanced by sex and obesity class while also maintaining relatively equal group sizes throughout the rolling recruitment period. The randomization sequence was generated using PASS statistical software (NCSS, LLC) by the study statistician (KAP). All other members of the research team, including staff, were blinded to the predetermined allocation sequence. The participant was then informed of their study group assignment and provided a Fitbit Versa 2, along with wear instructions for the 6-month intervention. Next, the research staff assisted the participant with downloading and installing the commercial Fitbit app onto their mobile phone, creating a study-specific email account to register with Fitbit for use during the study, and connecting their Fitbit account to the Fitabase data management platform to enable the research team to continuously collect PA data throughout the intervention. At the end of the baseline study session, participants were provided the study handbook and scheduled for their initial action planning session, which occurred at least 1 week after the baseline appointment, allowing time for the participant to become familiar with the Fitbit and the research team to collect the initial PA data necessary to inform the week 1 MVPA goal for participants assessed to the incremental study group. Participants then progressed through the 6-month intervention.

To assess longitudinal changes in self-regulation and self-efficacy over the duration of the intervention, participants were emailed a REDCap survey link to complete these psychosocial surveys each month during the intervention. After the 6-month intervention period, participants returned to the research center for their follow-up study assessment. These assessment procedures were similar to baseline, with the exception that participants were invited to complete a postintervention satisfaction survey to provide feedback on their overall experiences with the intervention, including what they liked and did not like about the intervention, specific challenges faced, and how we can refine our intervention efforts for a subsequent larger-scale trial. Participants were again given an ActiGraph GT9X Link accelerometer to wear for 7 days to objectively measure their PA levels, provided mailing materials to return the device after the 7-day wear period, and subsequently scheduled for a final Zoom session, in which the 7-Day Physical Activity Recall [44] was administered. Participants were also guided through the steps of connecting their Fitbit to their personal email address. Due to the nature of the behavioral intervention, study investigators, staff delivering the intervention, and participants were not blinded to study group assignment. However, all non–self-reported outcome data were collected by assessors blinded to the participants’ study group allocation.

### Measures

#### Sociodemographic Characteristics

Participant sociodemographic characteristics were assessed using a self-report questionnaire. These characteristics included age, sex, race/ethnicity, relationship status, income, education, and employment status.

#### Feasibility of Implementation

Feasibility was assessed by evaluating the research team’s ability to recruit, retain, and engage participants in the intervention; deliver the intervention as planned; and collect outcome data for the key measures identified for inclusion in a future larger-scale trial. Given the early stage of the research and following the recommendations of others [51], we did not set specific criteria or strict thresholds for establishing feasibility based on the individual metrics assessed. Instead, we adopted a holistic approach that focused on evaluating each feasibility metric within the context of the other metrics, with the goal of guiding protocol refinement for progression to larger-scale proof of concept trial (ie, stage 1B trial).

##### Participant Recruitment and Retention

Feasibility of recruitment was examined by tracking participant interest in the study via completion of the online eligibility screening, number of individuals eligible for participation based on completion of eligibility screening procedures, and the enrollment rate among those eligible. These data were collected to inform recruitment strategies and timelines for the subsequent larger-scale trial. Retention was calculated as the proportion of participants who completed the 6-month study assessment out of the total number of participants randomized.

##### Participant Engagement and Feasibility of Intervention Delivery

Participant engagement and feasibility of intervention delivery were assessed by examining the average number of coaching sessions each participant completed and the average number of days during the 6-month intervention period participants achieved valid Fitbit wear time, which was defined as wearing the Fitbit for at least 10 hours per day as determined by minute-level heart rate data. Periods without heart rate data were classified as nonwear during data processing.

##### Collection of Outcome Data

This outcome evaluated the ability and consistency of the research team to collect valid data from study participants for the key measures proposed for inclusion in a subsequent larger-scale trial of the intervention. Table 4 provides a brief description of each of these measures.

### Acceptability

Participants’ perceptions of and satisfaction with the intervention were assessed with a postintervention treatment acceptance survey administered at the end of the 6-month intervention. This 24-item measure, adapted from our previous work [52], included both multiple-choice and open-ended questions to capture participants’ overall perceptions of the intervention, as well as their thoughts on the specific components, including the action planning sessions and use of the Fitbit to track and self-monitor their MVPA. Example items assessing overall perceptions of the intervention included: “Overall, how helpful did you find the study for promoting physical activity?” and “How motivated were you to increase your physical activity as a result of the study?” Items targeting specific intervention components included: “How helpful did you find the physical activity coaching sessions for promoting physical activity?” “Do you think you gained knowledge about exercise and physical activity from your coaching session?” and “How helpful did you find using the Fitbit to help increase your activity?”

### Statistical Analysis

Quantitative data, including participant sociodemographic characteristics, intervention delivery and adherence rates, data collection rates, and Likert-like responses on the satisfaction survey, were summarized using descriptive statistics (eg, frequencies, means, and SDs). Fitbit wear time was determined using minute-level heart rate data; days or periods without recorded heart rate data were classified as nonwear time for analysis. Consistent with best practices for the design and evaluation of pilot studies [26,30,31], no formal hypothesis testing or inferential statistical analyses were performed, as the study was not sufficiently powered or intended to detect statistically significant effects. Qualitative data collected through the postintervention satisfaction survey were analyzed using direct content analysis [53]. For this analysis, participants’ responses to the open-ended questions were coded using a deductive approach, with the specific intervention components (eg, action planning sessions and Fitbit activity monitor) and a general category labeled “overall perceptions of the intervention” serving as predefined codes. Within each of these primary codes, participant narratives were further examined to identify recurring patterns and themes by 2 members of the research team (RPJ and MM) using a collaborative, iterative process. Final interpretation and reporting of key qualitative findings were achieved through consensus between the 2 analysts.

## Results

### Participant Characteristics

Participants (n=24) had a mean age of 54.1 (SD 5.8) years and a mean BMI of 36.3 (SD 5.0) kg/m^2^. The majority were female (21/24, 87.5%), identified as White (22/24, 91.6%) and non-Hispanic (21/24, 87.5%), and had a bachelor’s degree or higher (18/24, 75%). Table 5 provides participant sociodemographic characteristics.

**Table 5.** Sociodemographic characteristics of study participants (N=24).

Variable	Mean (SD)	Frequency, n (%)
Age (years)	54.1 (5.8)	N/A^a^
BMI	36.3 (5.0)	N/A
Sex		
Female	N/A	21 (87.5)
Male	N/A	3 (12.5)
Race		
White	N/A	22 (91.6)
American Indian or Alaskan	N/A	1 (4.2)
More than 1 race	N/A	1 (4.2)
Ethnicity		
Hispanic, Latino, or Spanish origin	N/A	3 (12.5)
Non-Hispanic, Latino, or Spanish origin	N/A	21 (87.5)
Relationship status		
Married	N/A	13 (54.2)
Not married but living with someone in a marriage-like relationship	N/A	2 (8.3)
Divorced	N/A	2 (8.3)
Never married	N/A	6 (25)
Declined to respond or missing	N/A	1 (4.2)
Annual household income		
<$25,000	N/A	1 (4.2)
Between $25,000 and $50,000	N/A	1 (4.2)
Between $50,001 and $75,000	N/A	5 (20.8)
Between $75,001 and $100,000	N/A	3 (12.5)
>$100,000	N/A	10 (50.0)
Declined to respond or missing	N/A	2 (8.3)
Education		
Two years of college or associate’s degree	N/A	4 (16.7)
Three years of college	N/A	2 (8.3)
Bachelor’s degree	N/A	10 (41.7)
Master’s degree	N/A	8 (33.3)
Employment status		
Employed full-time	N/A	17 (70.8)
Employed part-time	N/A	3 (12.5)
Unemployed	N/A	1 (4.2)
Homemaker	N/A	1 (4.2)
Declined to respond or missing	N/A	2 (8.3)

a N/A: not applicable.

### Feasibility of Implementation

#### Recruitment and Retention

**Figure 2.**
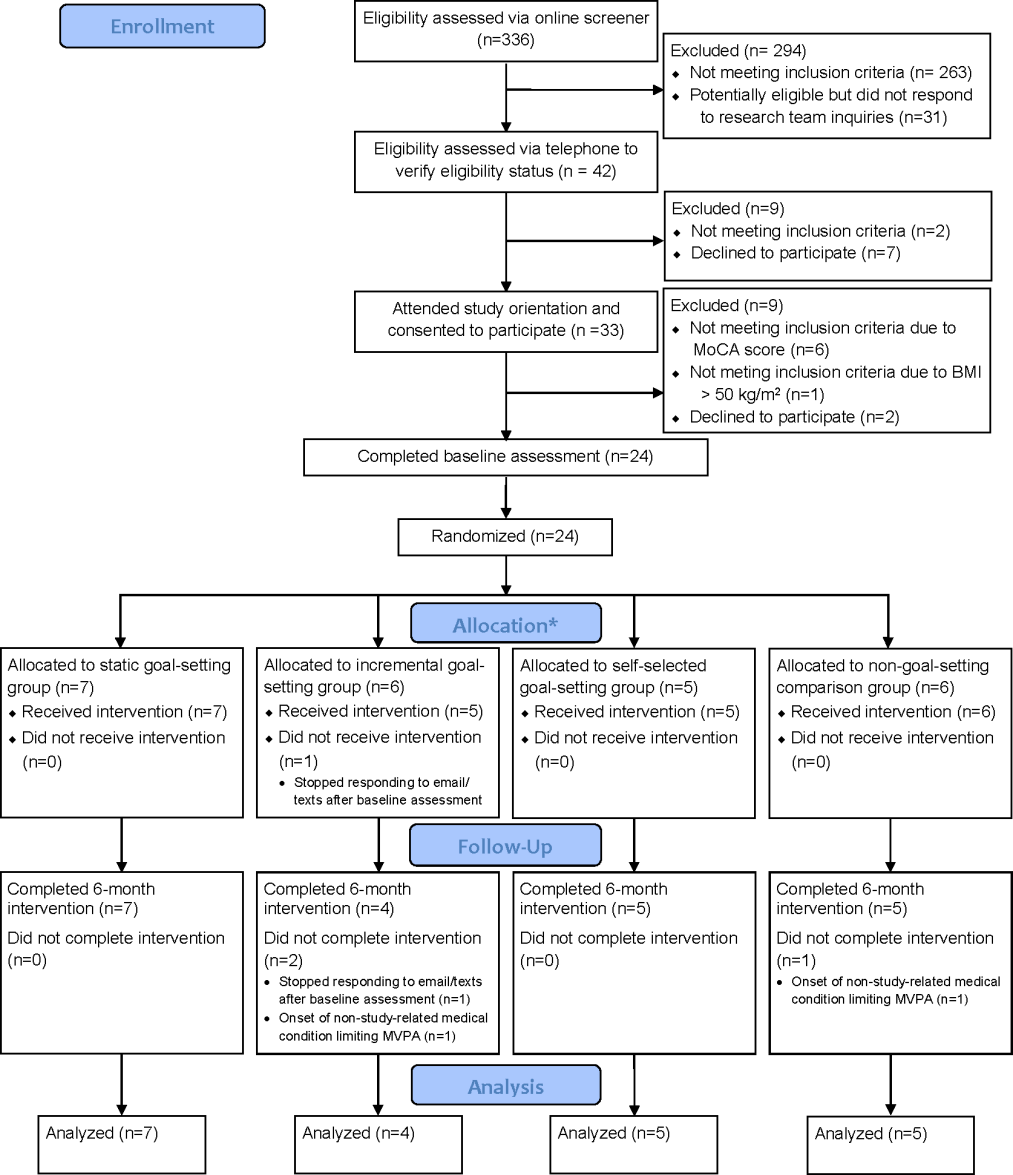
CONSORT (Consolidated Standards of Reporting Trials) diagram illustrating participant flow through recruitment, enrollment, randomization, and 6-month follow-up. MoCA: Montreal Cognitive Assessment; MVPA: moderate to vigorous physical activities. *Randomization error by research staff resulted in 7 participants being allocated to receive the static intervention group and 5 participants allocated to the self-selected group, instead of 6 in both of these study groups.

Figure 2 illustrates participant flow through the intervention. The sample of 24 study participants was enrolled in approximately 4 months (July 2024 through mid-October 2024), equating to enrolling 6 participants per month into the intervention. Recruitment efforts yielded 336 individuals completing the online eligibility screener, with 73 classified as potentially eligible based on online screening responses. Among these, 33 attended an in-person study orientation session where informed consent was obtained, BMI was verified, and the MoCA and the Center for Epidemiologic Studies Depression Scale were administered. This screening process resulted in 1 individual being excluded for study participation based on BMI status (ie, >50 kg/m^2^) and 6 individuals being excluded based on their MoCA scores. In addition, 2 participants declined study participation following the orientation, indicating that they were no longer interested in the study.

Among the 24 participants randomized to an intervention arm, 87.5% (n=21) were retained at 6 months. Reasons participants were lost to follow-up included the onset of a medical condition unrelated to study participation that limited participants’ ability to safely engage in MVPA (n=2) and loss of contact (n=1; ie, participant’s phone disconnected during coaching session 1 and subsequent attempts to reach the participant were successful).

### Intervention Delivery

#### Virtual Action Planning Sessions

Participants (24/24, 100%), on average, attended 14.6 out of 17 (85.9%) virtual action planning sessions. Eight participants (8/24, 33.0%) attended all 17 sessions. Among the 21 individuals completing the intervention, the average number of sessions attended was 15.9 out of the 17 (93.5%). Multimedia Appendix 1 illustrates action planning session attendance by study group over the duration of the 6-month intervention. Although the small sample size precluded formal testing of between-group differences in coaching session attendance, descriptive and visual analyses show similar patterns of session attendance across study groups and over time.

The duration of coaching sessions 1 and 2 (ie, more in-depth introduction, review of goals, and barriers) was mean 45.8 (SD 14.3) minutes and mean 39.9 (SD 11.6) minutes, respectively. Coaching sessions 3‐17 (ie, the brief follow-up coaching sessions) were mean 13.5 (SD 6.2) minutes in length. The majority of coaching sessions were conducted via telephone (175/351, 49.9%), followed by Zoom video call (172/351, 49.0%) and Zoom audio-only call (4/351, 1.1%).

#### Fitbit Versa 2 to Self-Monitor MVPA

Participants completing the study (n=21) received the coaching intervention over a mean of 180 (SD 11.3) days (exact duration of intervention delivery varied slightly based on participant availability for their 6-month outcome assessment) and provided valid Fitbit wear time (defined as wearing the device for >10 hours per day) on 87.1% (SD 15.8%) of these days (equating to approximately 156.7 days or 6.1 days per week). Average wear time for days meeting the 10 hours per day threshold for valid wear was a mean of 20.1 (SD 4.7) hours per day.

#### Collection of Outcome Data

Data collection rates for key outcomes identified for inclusion in a future larger-scale mechanistic trial were high, ranging from 95% to 100% at both the baseline and 6-month assessment periods (Table 6). Completion rates of monthly surveys assessing self-efficacy and self-regulation ranged from 91% to 100% among the 21 participants completing the study. Specific completion rates for surveys were 100% for months 1 and 2, 95% (20/21 participants) completion for month 3, 91% (19/21 participants) completion for month 4, and 95% (20/21 participants) completion for month 5. As illustrated in Multimedia Appendix 2, no clear pattern of differential survey completion emerged across study groups.

**Table 6.** Collection rates of valid assessment data at baseline and 6 months by study outcome and timepoint.

Measures	Baseline, n (%)	6 months, n (%)^a^
Self-regulation		
PA^b^ Self-Regulation Scale (PASR-12) [21]	24 (100)	21 (100)
PA Self-Regulation Scale from the Health Belief Survey [36]	24 (100)	21 (100)
Short Self-Regulation Questionnaire [38]	24 (100)	21 (100)
Self-efficacy		
Exercise Self-Efficacy Scale [40]	24 (100)	21 (100)
Self-Efficacy Scale [42]	24 (100)	21 (100)
Physical activity		
Accelerometer-Measured PA^c^	24 (100)	20 (95)^d^
7-Day PA Recall [44]	24 (100)	21 (100)
Cognitive functioning		
Memory	24 (100)	21 (100)
Executive Functioning	24 (100)	21 (100)
Processing Speed	24 (100)	21 (100)
ADRD^e^ and cardiometabolic disease risk		
Phlebotomy procedures for serum and plasma measures of ADRD and cardiometabolic disease risk	23 (96)^f^	20 (95)^f^
Cardiorespiratory Fitness assessed using a modified Balke treadmill test	23 (96)^g^	21 (100)
Body weight and anthropometrics	24 (100)	21 (100)
Blood pressure	24 (100)	21 (100)

a Percentages at the 6 months were calculated based on the number of participants completing the intervention (n=21).

b PA: physical activity.

c Participants, on average, wore the ActiGraph GT9X+ for approximately 16.3 (1.97) waking hours per day on 6.6 (0.82) valid days per week at baseline and 16.1 (1.88) waking hours per day on 6.8 (0.53) valid days per week at 6 months.

d One participant reported having a wrist injury that limited the use of wrist-worn device on their nondominant wrist and thus was not asked to wear the ActiGraph device.

e ADRD: Alzheimer disease and related dementias.

f Phlebotomists were unable to draw blood from the same participant at both baseline and 6-month assessment periods due to the inability of locating a vein suitable for the blood draw procedure.

g One participant’s test was terminated before reaching peak oxygen consumption (VO_2peak_) due to the participant feeling lightheaded.

### Acceptability

#### Action Planning Sessions

**Figure 3.**
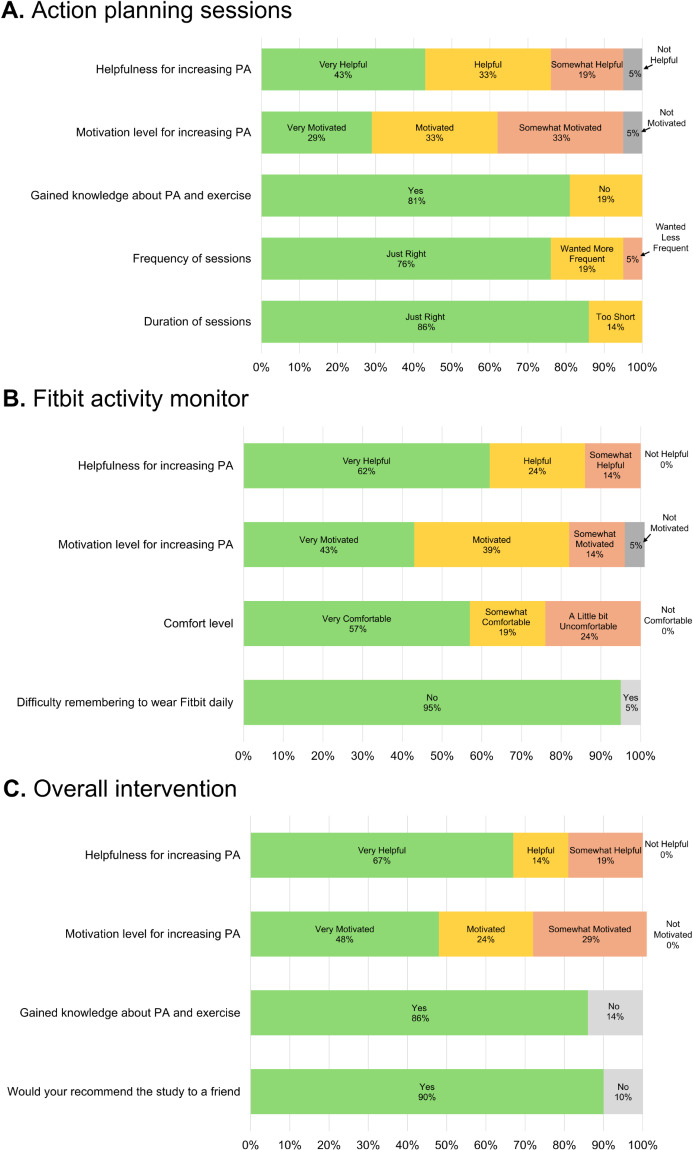
Participant-reported acceptability of the action planning sessions, Fitbit activity monitor, and overall intervention. (A) Acceptability ratings for the action planning sessions. (B) Acceptability ratings for the Fitbit activity monitor. (C) Acceptability ratings for the overall intervention. PA: physical activity.

Participants reported high satisfaction with the action planning sessions. Among the 21 participants completing the intervention, most participants rated the sessions as “very helpful” (n=9/21, 43%) or “helpful” (n=7/21, 33%) for increasing their PA (Figure 3A in Figure 3A, ). Four participants (4/21; 19%) reported that the action planning sessions were “somewhat helpful,” and 1 participant reported that they were “not helpful at all.” Likewise, most participants found the action planning sessions to be motivating for increasing PA (n=6/21, 29% “very motivated”; n=7/21, 33% “motivated”; and n=7/21, 33% “somewhat motivated”; Figure 3A). Only 1 participant indicated that they were “not motivated” by the sessions. Additionally, 81% (n=17/21) of the participants reported gaining knowledge about PA and exercise from the coaching sessions.

Regarding the frequency and duration of the action planning sessions, participants generally reported favorable responses. Seventy-six percent (16/21) of the participants completing the study reported that the frequency of sessions was “just right,” while 19% (4/21) reported the desire for more frequent sessions and 1 participant reported that the coaching sessions were too frequent. Similarly, most participants were satisfied with the length of the coaching sessions, with 86% (18/21) reporting that the duration of the coaching sessions was the “right amount of time,” whereas 14% (3/21) reported that the sessions were “too short”; no participants reported that the sessions were too long.

Participant narratives describing their experiences with the action planning sessions largely aligned with the positive responses from the closed-ended satisfaction survey. Many participants shared that they enjoyed the sessions, as well as the interventionists delivering them, and found the sessions to be encouraging for increasing their PA. Qualitative narratives highlighting this included, “[Coach’s name] was a joy...the length and frequency [of the sessions] were pretty good. She met me where I was at.,” “Coach was very personable and positive,” “She [coach] was encouraging when I was discouraged,” “It kept me accountable.,” “Helped me think more about being active and ways to do it,” and “Early in the study week, goal setting really helped me to be mindful and achieve my goals.*”*

When asked how we can improve the coaching sessions, approximately half of the narratives provided by participants (n=8 out of 18) indicated that either no change was needed (n=4) or expressed satisfaction without offering specific suggestions for improvement (n=4). Quotes illustrating this included, “I don’t sense improvement is needed.,” “No changes.,” “I felt the sessions were conducted very well,” and “I was happy with the coaching.” However, among participants offering constructive feedback, a common theme was the desire for more variety and flexibility in the coaching sessions. Some participants felt that the sessions were somewhat repetitive and suggested revisiting key topics from the initial action planning sessions (eg, sources of social support and short- and long-term goals) in the later sessions. Others emphasized the need for greater adaptability based on individual needs. Quotes illustrating these sentiments included, “[coaching sessions] never followed up on the [long or short-term] goals so there was not accountability.,” “Sessions felt very stagnant, not personalized...the goals/rewards that were set in the beginning were never visited again,” “I did not find revisiting my [weekly] goals every session was helpful,” and “The sessions were too scripted. More flexibility to meet the needs of the participants would be nice.*”*

#### Fitbit Activity Monitor

Most participants rated the Fitbit as “very helpful” (13/21, 62.0%) or “helpful” (5/21, 24.0%) for increasing their PA. Three participants (3/21, 14%) rated the device as being “somewhat helpful”; no participants rated it as being “not helpful” (Figure 3B). When asked how motivated they were to increase their PA as a result of wearing the Fitbit, the majority shared that they were either “very motivated” (9/21, 43%) or “motivated” (8/21, 38%). Three participants out of 21 (14%) reported that they were “somewhat motivated”; 1 participant out of 21 (5%) reported that they were “not motivated.” Additionally, most participants found the Fitbit to be comfortable to wear (n=12/21 “very comfortable”; n=4/21 “somewhat comfortable”; and n=5/21 “a little bit uncomfortable) and the overwhelming majority reported not experiencing any difficulties remembering to wear the Fitbit daily (20/21, 95%).

Although most feedback regarding the Fitbit was favorable, some participants expressed discontent with the device during the intervention, as well as on the postintervention satisfaction survey. These concerns were both general in nature and specific in regard to the perceived accuracy of the Fitbit for assessing MVPA. Quotes illustrating general concerns included, “Wish the Fitbit would integrate better with iPhone.,” “The Fitbit kinda sucks now (used to be better),” and “Maybe an alternative to the Fitbit.” Accuracy-related concerns centered on the perception that the device overreported MVPA, with 1 participant noting during a coaching session that she was accumulating Active Zone Minutes while sitting in traffic. Although much of these discussions regarding the accuracy of the Fitbit occurred during structured coaching sessions, 1 participant mentioned this on the postintervention focus group with the following, “Fitbit was very inaccurate in regard to activity minutes.” Notably, this concern was also shared by the research team during intervention delivery, as Fitbit data collected for some participants continually reported elevated levels of MVPA.

#### Overall Feedback About the Intervention

Overall feedback regarding the acceptability of the intervention generally reflected findings of the individual components. For example, most participants found the intervention to be “very helpful” (14/21, 67%) or “helpful” (3/21, 14%) for promoting PA and indicated that they were either “very motivated” (10/21, 48%) or “motivated” (5/21, 24%) to increase their PA as a result of the study (Figure 3C). In addition, 86% (18/21) reported gaining knowledge about PA and exercise from the study, and 90% (19/21) reported that they would recommend the study to a friend.

When asked how we can improve the intervention for larger-scale testing, almost half of the responses to this open-ended question (6/15 responses, 40%) either reflected that no changes were needed or offered only positive feedback. Illustrative comments included, “No improvements needed,” “Nothing,” and “The program was great. Sorry for not being able to suggest any improvements.” In addition, 3 participants shared that they would like an in-person component incorporated into the study to allow for group exercise and/or instruction of how to perform certain activities. These quotes included, “I would not mind an in-person exercise class or 2 or more specific guidance on skills” and “Offer workshops like beginners pickleball or basketball shooting.” The remaining responses to this item were varied and did not reveal insights not previously reported.

## Discussion

### Principal Results

This study examined the feasibility and acceptability of a newly developed intervention designed to compare 3 different goal-setting approaches for enhancement of PA-related self-regulation and self-efficacy and adherence to national PA guidelines. The results demonstrated promising outcomes for advancing this line of research in a larger-scale trial, as evidenced by high participant retention and engagement, fidelity of intervention delivery, successful collection of primary outcome measures, and high participant satisfaction. Findings also identified several areas for refinement prior to broader implementation.

### Interpretation of Findings and Comparisons With Prior Work

The research team was able to successfully recruit the study sample size in approximately 4 months, yielding a recruitment rate of approximately 6 participants per month. Given this study served as the research team’s first attempt to recruit midlife adults with obesity using the specific inclusion criteria and eligibility screening procedures of this study, this outcome provides valuable insight for developing recruitment timelines for future studies for our intended study population. The observed retention rate of 88% was comparable with, or slightly higher than average rates reported in reviews and meta-analyses of health behavior change trials of similar durations [54], including PA interventions [12,55], lending support for feasibility of the intervention approach. Similarly, adherence to the action planning sessions was high, with participants attending 85.9% of sessions, as was Fitbit compliance, with valid wear (>10 hours per day) achieved on 87.1% of days. Successful collection of outcome data for measures intended for inclusion in a future larger-scale trial was also high, with valid data obtained for 91%-100% of participants across measures.

Participant acceptability of the intervention was favorable, as most participants found the overall intervention, as well as the individual components (ie, action planning sessions and Fitbit activity monitor) to be useful and motivating for increasing PA. However, qualitative narratives provided by participants highlighted several areas in which the intervention could be refined in future research. For example, several participants noted repetitiveness of some of the action planning session content, as well as the desire for follow-up on key topics discussed during the initial sessions. This feedback suggested the need to revise certain sessions to reduce redundancy and to enhance the brief action planning components by incorporating more structured follow-up on the comprehensive action plans developed in sessions 1 and 2. Participant concerns regarding the accuracy of the Fitbit for assessment of MVPA were also shared by the research team, as the research staff observed some participants consistently receiving high levels of weekly Active Zone Minutes. In an effort to explore concerns regarding Fitbit’s accuracy, the research team informally compared minute-level heart rate data against minute-level Active Zone Minute data. Findings suggested that participants were accumulating Active Zone Minutes at a heart rate threshold of approximately 50%‐60% of their age-estimated maximum. Although this range aligns with recommendations for moderate-intensity PA from the American Heart Association [56], it is substantially below the 64% threshold recommended by the American College of Sports Medicine [57]. Given Fitbit’s algorithm for determining MVPA and awarding Active Zone Minutes is proprietary, the exact methods used to assess MVPA are not publicly available.

### Strengths and Limitations

This study has several strengths. The overarching 4-arm study design represents an experimental medicine approach to generate understanding of how goal setting influences the psychosocial processes of self-regulation and self-efficacy in promoting PA. The NIH Science of Behavior Change Program has encouraged such approaches to promote better understanding of *how* and *why* specific behavior change techniques lead to (or fail to produce) behavior change [20]. This study represents a necessary first step in pursuing this type of approach for understanding how different approaches to goal setting can increase PA and ultimately promote adherence to national PA guidelines. Second, selection of the primary outcome measures used to assess feasibility and acceptability, as well as the determination of the sample size, was guided by established frameworks [26] and guidelines [51] for conducting pilot and feasibility trials. Previous reviews and critiques of pilot and feasibility studies [30,31] have noted that many authors have inappropriately focused on outcomes related to efficacy or effectiveness, which are not suitable for feasibility studies. This study was carefully designed to avoid this common pitfall. Similarly, reporting of the study protocol and outcomes followed CONSORT guidelines [29] for randomized pilot and feasibility trials.

Several limitations should also be acknowledged. Although the goal-setting techniques included in this study are common approaches used in the PA promotion literature, they represent only a subset of ways in which goal setting can be operationalized. Numerous other approaches to conceptualizing and implementing PA goal setting exist, including variations in the underlying theoretical approach (eg, implementation intentions [58] and achievement goal theories [59,60]), PA metric used (eg, MVPA, steps, and frequency of PA sessions), and frequency of goal setting and evaluation of achievement. Exploring alternative approaches to PA goal setting is important for future research. In addition, despite a primary focus of the study being on the feasibility of intervention delivery, the action planning sessions were not audio recorded, limiting the ability to assess fidelity of the delivery of the session curriculum. Given that this is an important aspect to include for process evaluation, it will be included in future studies. Another potential limitation is that our study sample consisted of mostly non-Hispanic White women with higher income and education levels, which may limit generalizability of study outcomes. We anticipate that these sample characteristics are related to the use of convenience sample methods that primarily included social media and online advertising for recruitment. In future studies, we will prioritize more inclusive recruitment strategies to enhance the diversity of the study sample. Additionally, the validity of Fitbit’s proprietary measures of MVPA (ie, Active Zone and Active Zone Minutes) is not publicly known, limiting definitive understanding regarding whether the metric is a scientifically valid method for assessment of MVPA. Finally, collection of acceptability data was limited to completers of the 6-month intervention, which may have introduced selection and social desirability bias, potentially positively skewing these results. However, given the high retention rate, we anticipate such bias to be minimal.

### Conclusions and Implications for Future Research

Building on the findings from this pilot study, the research team implemented several modifications and refinements to the study protocol in preparation for a larger-scale testing. These refinements included (1) revising the action planning session curriculum to reduce repetitiveness and to include follow-up conversations regarding key topics discussed in the initial 2 action planning sessions (ie, social support, rewards, and short-term and long-term goals) in the latter, brief action planning sessions; (2) audio-recording of all action planning sessions to enable assessment of fidelity of session delivery; and (3) replacing the Fitbit Versa 2 with the Garmin Vivoactive 5 as the consumer wearable used by the participants to self-monitor their MVPA. The Garmin device allows for the research team to apply specific thresholds for classification of moderate- and vigorous-intensity activity (eg, 40% and 60% of participants’ heart rate reserve [57]), rather than relying on Fitbit’s proprietary algorithm.

The next step for this line of work is for our research team to expand upon these promising findings and to conduct a larger-scale (n=120) phase 1B proof-of-concept trial (based on National Institute on Aging’s Stage Model). This trial will examine the preliminary effects of the 3 goal-setting techniques, compared with the non–goal-setting comparison group, on enhancing self-regulation, self-efficacy, and adherence to national aerobic PA guidelines over a 9-month intervention period. This intervention will consist of a 6-month active intervention delivery phase followed by a 3-month minimal contact period, allowing for additional insight into whether enhancements in self-regulation, self-efficacy, and MVPA are maintained after the active intervention delivery ends. These preliminary findings will provide valuable data regarding the effectiveness of various goal-setting techniques to maintain behavior change over time that will support overall physical health as well as reduce risk for ADRD.

## Supplementary material

10.2196/82494 Multimedia Appendix 1 Frequency of action planning session attendance by study group. (A) Static weekly MVPA goal of 150 minutes (n=7) study group. (B) Self-selected weekly MVPA goal (n=5) study group. (C) Incremental weekly MVPA goal (n=4) study group. (D) Non–goal-setting comparison (n=5) study group. MVPA: moderate to vigorous physical activity.

10.2196/82494 Multimedia Appendix 2 Frequency of participant completion of monthly surveys assessing self-regulation and self-efficacy by study group. (A) Static weekly MVPA goal of 150 minutes (n=7) study group. (B) Self-selected weekly MVPA goal (n=5) study group. (C) Incremental weekly MVPA goal (n=4) study group. (D) Non–goal-setting comparison (n=5) study group. MVPA: moderate to vigorous physical activities.

## Abbreviations

ADRD: Alzheimer disease and related dementias
CONSORT: Consolidated Standards of Reporting Trials
MoCA: Montreal Cognitive Assessment
MVPA: moderate to vigorous physical activity
NIH: National Institutes of Health
PA: physical activity
REDCap: Research Electronic Data Capture
SCT: Social Cognitive Theory
