# Supplementary figures and images for: Comparing 3 Goal-Setting Techniques to Promote Adherence to National Physical Activity Guidelines in Midlife Adults: Feasibility Trial of a Mechanistic Study Design
Source: JMIR Form Res. 2026 Mar 16;10:e82494. doi: 10.2196/82494 (PMC12991197; doi:10.2196/82494)

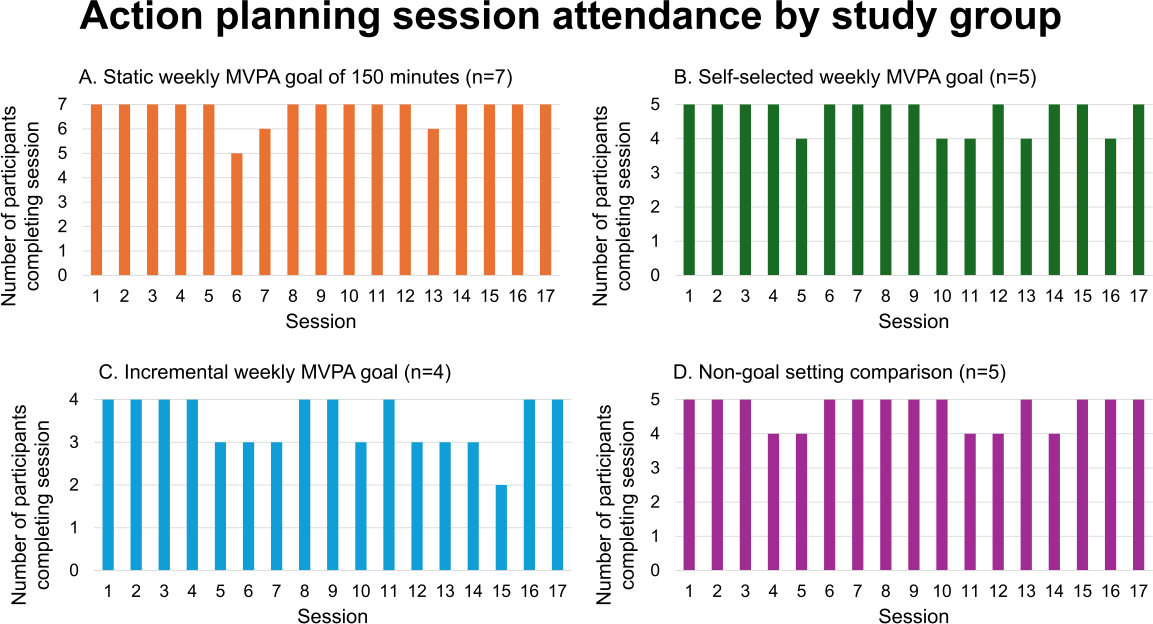

Supplement: Multimedia Appendix 1 [file formative-v10-e82494-s001.png]

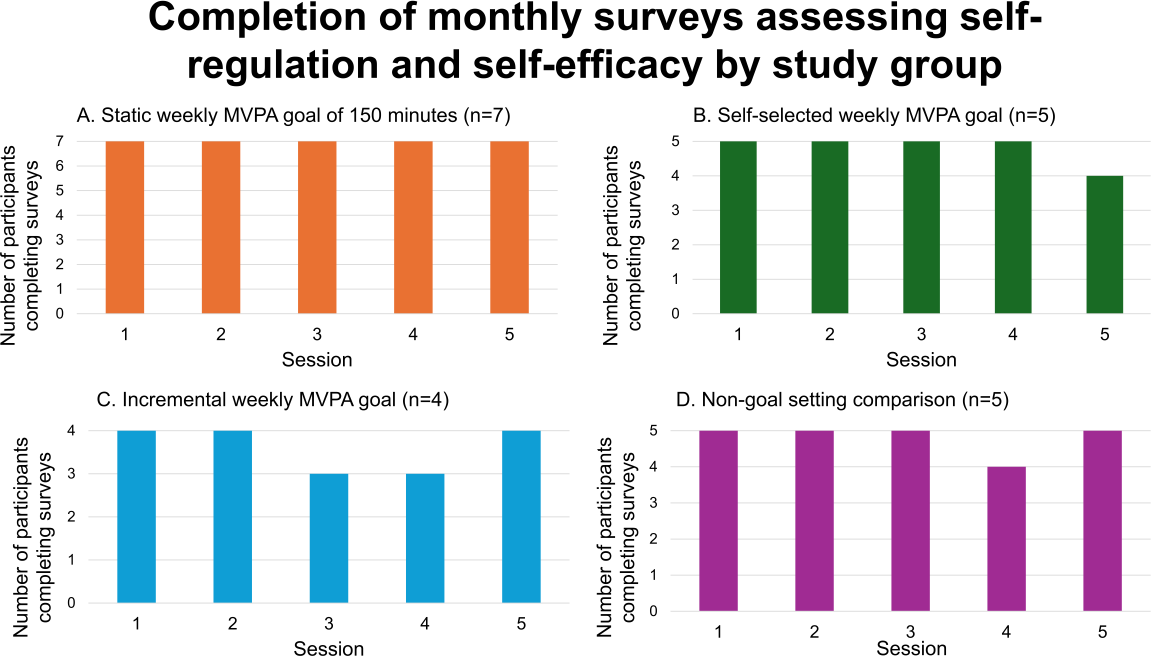

Supplement: Multimedia Appendix 2 [file formative-v10-e82494-s002.png]
